# CRTC2 activates the epithelial–mesenchymal transition of diabetic kidney disease through the CREB-Smad2/3 pathway

**DOI:** 10.1186/s10020-023-00744-0

**Published:** 2023-10-26

**Authors:** Yujie Li, Yufeng Zhang, Hongshuo Shi, Xuemei Liu, Zifa Li, Jiayi Zhang, Xiuge Wang, Wenbo Wang, Xiaolin Tong

**Affiliations:** 1 Changchun University of Traditional Chinese Medicine, Changchun, 130012 China; 2 College of Traditional Chinese Medicine, Shandong University of Traditional Chinese Medicine, Jinan, 250000 China; 3 The Second Affiliated Hospital of Shandong University of Traditional Chinese Medicine, Jinan, 250000 China; 4 Experimental Center, Shandong University of Traditional Chinese Medicine, Jinan, 250000 China; 5 The First Affiliated Hospital of Changchun University of Chinese Medicine, Changchun, 130012 China; 6 Department of Endocrinology, Guang’anmen Hospital, China Academy of Chinese Medical Sciences, Beijing, China

**Keywords:** Epithelial-to-mesenchymal transition, Diabetic kidney disease, CTRC2, CREB, Smad2/3

## Abstract

**Background:**

Epithelial–mesenchymal transition (EMT) plays a key role in tubulointerstitial fibrosis, which is a hallmark of diabetic kidney disease (DKD). Our previous studies showed that CRTC2 can simultaneously regulate glucose metabolism and lipid metabolism. However, it is still unclear whether CRTC2 participates in the EMT process in DKD.

**Methods:**

We used protein‒protein network (PPI) analysis to identify genes that were differentially expressed during DKD and EMT. Then, we constructed a diabetic mouse model by administering STZ plus a high-fat diet, and we used HK-2 cells that were verified to confirm the bioinformatics research results. The effects that were exerted by CRTC2 on epithelial-mesenchymal transition in diabetic kidney disease through the CREB-Smad2/3 signaling pathway were investigated in vivo and in vitro by real-time PCR, WB, IHC and double luciferase reporter gene experiments.

**Results:**

First, bioinformatics research showed that CRTC2 may promote EMT in diabetic renal tubules through the CREB-Smad2/3 signaling pathway. Furthermore, the Western blotting and real-time PCR results showed that CRTC2 overexpression reduced the expression of E-cadherin in HK-2 cells. The CRTC2 and α-SMA levels were increased in STZ-treated mouse kidneys, and the E-cadherin level was reduced. The luciferase activity of α-SMA, which is the key protein in EMT, was sharply increased in response to the overexpression of CRTC2 and decreased after the silencing of CREB and Smad2/3. However, the expression of E-cadherin showed the opposite trends. In the real-time PCR experiment, the mRNA expression of α-SMA increased significantly when CRTC2 was overexpressed but partially decreased when CREB and Smad2/3 were silenced. However, E-cadherin expression showed the opposite result.

**Conclusion:**

This study demonstrated that CRTC2 activates the EMT process via the CREB-Smad2/3 signaling pathway in diabetic renal tubules.

**Supplementary Information:**

The online version contains supplementary material available at 10.1186/s10020-023-00744-0.

## Background

Diabetic kidney disease (DKD, as well as DN) is a refractory chronic microvascular complication of diabetes that eventually develops into end-stage renal disease (ESRD) (Sun et al. 2013), and it occurs in 40% of patients with type 2 diabetes (Thomas et al. 2015). The pathological changes that occur in DKD include kidney hypertrophy and extracellular matrix accumulation, which lead to glomerular sclerosis and tubular interstitial fibrosis (Sha et al. 2020). Clinical studies have shown that tubulointerstitial fibrosis occurs earlier than glomerular fibrosis and is more closely related to renal prognosis during the development of DKD (Mise et al. 2016). In the past, most studies on DKD have focused on glomerular lesions, and there has been very limited research on the occurrence and development, as well as the underlying mechanisms, of renal tubulointerstitial lesions, which account for almost 90% of the entire kidney volume under diabetic conditions. Recently, many studies have shown that renal tubulointerstitial changes (such as fibrosis) seem to be essential for the progression of DKD to ESRD (Xu et al. 2020).

Epithelial-to-mesenchymal transition (EMT) is an important factor that contributes to DKD, leading to tubular interstitial fibrosis and even ESRD. During EMT in DKD, proximal tubular epithelial cells lose apical polarity and E-cadherin expression and adopt a myofibroblast phenotype via the expression of α-smooth muscle actin (α-SMA) (Li et al. 2020). It has been found that epithelial cells in renal fibrotic tissues are involved in the process of EMT, and inhibition of the EMT of epithelial cells can prevent chronic kidney damage and fibrosis (Li et al. 2020; Xu et al. 2020).

Cyclic adenosine monophosphate-responsive element (CRE) binding protein–regulated transcription coactivator (CRTC) 2 is a new cAMP response element-binding (CREB) protein coactivator that was discovered in 2003 almost simultaneously by two research teams using genomic high-throughput screening (Conkright et al. 2003; Iourgenko et al. 2003), and extensive research has been conducted on glucose homeostasis (Koo et al. 2005). CRTC2 is involved in regulating insulin sensitivity, endoplasmic reticulum stress (Wang et al. 2010b), endoplasmic reticulum stress(Wang et al. 2009), viral activation, and obesity(Murata et al. 2009), and CRTC2 plays an important role in regulating liver cell gluconeogenesis and maintaining the stability of fasting blood glucose. In fasting animals, the glucagon signal increases and dephosphorylates CRTC2 (Li et al. 2017b), which then is translocated from the cytoplasm to the nucleus, where it binds to phosphorylated CREB and activates gluconeogenesis (Zhang et al. 2018). The diabetes control and complications trial (DCCT) (Lachin et al. 2017) and UK diabetes prospective study (UKPDS) (Zoungas et al. 2017) have proven that strict control of blood glucose can significantly reduce the occurrence of microalbuminuria or progression to nephropathy. Therefore, CRTC2, which is an important regulator of glucose metabolism, may play a role in the occurrence and development of diabetic kidney disease. However, no in-depth study on the role of CRTC2 in DKD has been reported to date, which prompted us to investigate whether CRTC2 affects the DKD process.

We used computational tools to simulate and predict the disease mechanism and biological process and provide evidence to support the next specific experiment (Mangul et al. 2019). In this study, we used the Gene Expression Omnibus (GEO) database, EMT-related database, and STRING database to predict the potential molecular mechanism by which CRTC2 affects EMT in DKD patients. Furthermore, through a series of studies in mouse and cellular models, it was demonstrated that CRTC2 promoted tubulointerstitial fibrosis in DKD.

## Materials and methods

### Data processing

We downloaded EMT-related genes (ERGs) from the molecular feature database v7.1 (http://www.broadinstitute.org/gsea/msigdb/index.jsp) and the EMT gene database (http://dbemt.bioinfo-minzhao.org/download.cgi). All the EMT-related genes are listed in Additional file 1. The GEO (http://www.ncbi.nlm.nih.gov/geo) database serves as a public genomics database that contains a large amount of gene expression data (Edgar et al. 2002). We downloaded a dataset, namely, GSE142153, from the GEO. This dataset contains the transcription profiles of PBMCs from DKD patients and control groups, and this dataset includes 10 healthy samples and 23 DKD samples.

### Differentially expressed ERGs and protein–protein network (PPI) analysis

We identified differentially expressed genes (DEGs) in the expression data of the DN microarray through the limma software package in R software (P < 0.05). We mapped DEGs with ERGs to obtain the DEGs from among the ERGs in DN, and the gene set was defined as ERGs-DN. Then, we imported the ERGs-DN gene set into the STRING database (http://string-db.org, version 11.0) (combined score > 0.4), and the STRING database was utilized to predict PPI networks, which can further explain the mechanism underlying disease occurrence and development (Szklarczyk et al. 2015). Subsequently, we imported the PPI network into Cytoscape 3.7.2 and used the plug-in MCODE (Molecular Complexity Detection) which is used to cluster networks based on topology, to determine densely connected areas in Cytoscape to analyze the core modules of the PPI network. Then, we imported CRTC2 and the core module into the STRING database to determine its potential relationship.

### Animals and treatment

C57BL/6J mice (6–7 weeks, male) were used in the experiments. After obtaining the experimental mice, they were adaptively reared in the clean animal room of Shandong University of Traditional Chinese Medicine for 7 days. The mice were allowed to drink freely and were reared under standard experimental conditions: temperature 25 ± 2 °C, humidity 60 ± 5%, day and night cycle 12/12 h.

The mice were divided into two groups: control mice were fed a normal diet, and STZ-treated mice were fed a high-fat diet (16% fat, 20% sucrose and 3% yolk powder) for two weeks and then injected with STZ (30 mg/kg/d) intraperitoneally daily for 5 days to establish a diabetic mouse model. We measured the blood glucose levels of the mice and determined that diabetic model mice had blood glucose levels > 11.1 mmol/L after injection for 5 days. We weighed the mice every two weeks for 12 weeks. A 24-h urine sample was collected, and its volume was measured before sacrifice. All the mice were fasted for 6 h before sacrifice. Two kidneys were removed: one was stored at − 80 °C (RNA and protein preparations), and the other was stored in 4% formalin for immunohistochemical and histological evaluation. Our research was specifically approved by the Ethics Committee of Shandong University of Traditional Chinese Medicine (Jinan, China). All the protocols were approved by the Animal Care and Use Committee of Shandong University of Traditional Chinese Medicine (Jinan, China), and the methods were performed according to the approved guidelines. All the surgical procedures were performed under sodium pentobarbital anesthesia, and all efforts were made to minimize suffering.

### Cell culture

HK-2 cells were purchased from the Chinese Academy of Science. HK-2 cells were cultured in Dulbecco’s modified Eagle medium supplemented with 10% fetal bovine serum, 100 U/ml penicillin, and 100 mg/ml streptomycin. Cells were grown to 80–90% confluence to treat according to experiments.

### Oral glucose tolerance tests

In order to assess the fasting blood glucose level of the mice, we fasted the mice overnight for 16 h, during which they were allowed to drink freely. In the oral glucose tolerance test (OGTT), we gave mice glucose (2 g/kg body weight), and then collected tail blood samples every 30 min to determine glucose levels.

### Small interfering RNA transfection

We purchased small interfering RNAs (siRNAs) targeting the human Smad2/3 (Santa Cruz Biotechnology) and CREB siRNAs (Cell Signaling Technology). We used lipofectamine 3000 reagent (Invitrogen company) to transfect siRNA according to the manufacturer's instructions. In 35-mm dishes, HK-2 cells were transfected with or without thirty pmol of Smad2/3 or CREB siRNA, with a corresponding volume of transfection reagent. After 48 h, the HK-2 cells were collected for RNA analysis.

### Plasmid transfection and reporter assay

Previously, we obtained the pCMV-CRTC2-flag plasmid described and provided by Dr. Jeffery L. Meier (Infectious Disease Department, Carver School of Medicine, University of Iowa). The plasmid contains human CRTC2 that is C-terminally tagged with Myc and Flag, and we used lipofectamine 3000 reagent (Invitrogen) for transfection analysis according to the manufacturer's instructions. For reporter gene detection, cells were co-transfected with 0.5 mg E-cadherin or α-SMA luciferase with or without CRTC2-Flag plasmids, CREB siRNA, or Smad2/3 siRNA, and the internal reference was the renilla luciferase plasmid pRL-SV40 (Promega, Madison, WI). After 48 h, we washed once with PBS and shake the culture plate in 1 × buffer (Promega) to harvest the cells. According to the manufacturer’s protocol, the dual-luciferase reporter detection system (Promega) was used to measure the activity levels of firefly and kidney cell luciferase in the cell lysate. The level of firefly luciferase activity in the lysate of transfected cells was normalized to the level of renal cell luciferase activity.

### H&E staining

We embed the removed kidney tissue in paraffin and slice it to 5 μm, and we used hematoxylin and eosin (H&E) to stain the sections and observe under an optical microscope.

### Co-immunoprecipitation assay and western blot analysis

For total protein extraction, cells were homogenized in RIPA buffer containing protease and phosphatase inhibitors. Protein concentrations were determined using the BCA method. Immunoprecipitation (IP) was then performed on 600 mg total protein from each treatment tube. Proteins were incubated overnight at 4 °C with normal mouse IgG (Beyotime Biotechnology, Inc.) and CBP antibody. The next day 30 ml pre-cleared Protein A/G Agarose (Beyotime Biotechnology, Inc.) was added to each treatment tube and spun with end-to-end rotation at 4 °C for 2 h. The Eppendorf tubes were then centrifuged, the supernatant was discarded, and the beads were washed with PBS buffer. Fifty microliters Laemmli Sample Buffer was then added to each treatment tube, after which the tubes were heated to 100 °C for 10 min and centrifuged, and Western blots were performed the supernatant. Equal aliquots (20 ml) from each treatment were separated on 10% SDS-PAGE gel and transferred onto a PVDF membrane (Millipore). The membranes were then probed with primary antibodies overnight at 4 °C followed by incubation with peroxidase-conjugated anti-rabbit or anti-mouse secondary antibodies (1:10,000) for 1 h at room temperature. After washing with 1xTBST, the bound primary antibodies (anti-α-SMA, E-cadherin, and anti-CRTC2 (Proteintech, inc), anti-p-Smad (Abcam) and anti-p-CREB antibody (cell signaling) were visualized with Alpha Q and exposed to film. The same membrane was stripped and reblotted with anti-GAPDH antibody (Cwbiotech, Beijing, China) as a loading control.

### Real-time quantitative PCR

Total RNA was isolated from cells and fresh tissues using an RNeasy Total RNA Isolation kit (TaKaRa) and was reverse transcribed into cDNA (TaKaRa). SYBR Green (DBI) quantitative PCR analysis reactions were then performed using the Roche 480 detection system. Each reaction consisted of 10 ml of SYBR green, 1 ml of cDNA, 1.0 ml of each primer pair (10 mmol/ml), and 8.0 ml of distilled water. The β-actin gene was simultaneously detected as a control. Relative gene expression levels were quantifified using the 2^−ΔΔ^Ct method, and the results were expressed as the fold change relative to the control. The primer sequences are as follows: CRTC2: (forward primer) AGTGCAGATGGTAGTCGAAACAA, (reverse primer) CGATACACCCGC-ACATTG; CREB: (forward primer) CCACTGTAACGGTGCCAACT, (reverse primer) GCTGCATTGGTCATGGTTAATGT; Smad3: (forward primer) CCACTACC-AGAGAGTAGAGAC, (reverse primer) GTTCATCTGGTGGTCACTGGT; α-SMA: (reverse primer) TTCAATGTCCCAGCCATGTA, (reverse primer) GAAGGAATAG-CCACGCTCAG; E-cadherin: (forward primer) TGGGTGAATTCGGGCTTGTT, (reverse primer) TGAAGGTGACAGAGCCTCTGGA; Vimentin: (forward primer) TG-CTTCAAGACTCGGTGGAC, (reverse primer) ATCTCCTCCTCGTACAGGTCG; β-catenin: (forward primer) CCCAGTCCTTCACGCAAGAG, (reverse primer) CATCT-AGCGTCTCAGGGAACA; Snai1: (forward primer) CACACGCTGCCTTGTGTCT, (reverse primer) GGTCAGCAAAAGCACGGTT; β-actin: (forward primer) ACAGA-GCCTCGCCTTTGC, (reverse primer) ACATGCCGGAGCCGTTGT.

### Statistical analysis

The results are presented as mean values ± standard deviation (Mean ± SD) for the number of experiments indicated. Statistical significance between two conditions was determined using the Student t test, while one-way analysis of variance (Dunnett t or least signifificant difference test) was used for multiple comparisons. Data were analyzed using SPSS 18.0 software. All test items in this study were repeated at least three times. Differences were considered significant at P < 0.05.

## Results

### Identification of ERGs in diabetic kidney disease

**Fig. 1:**
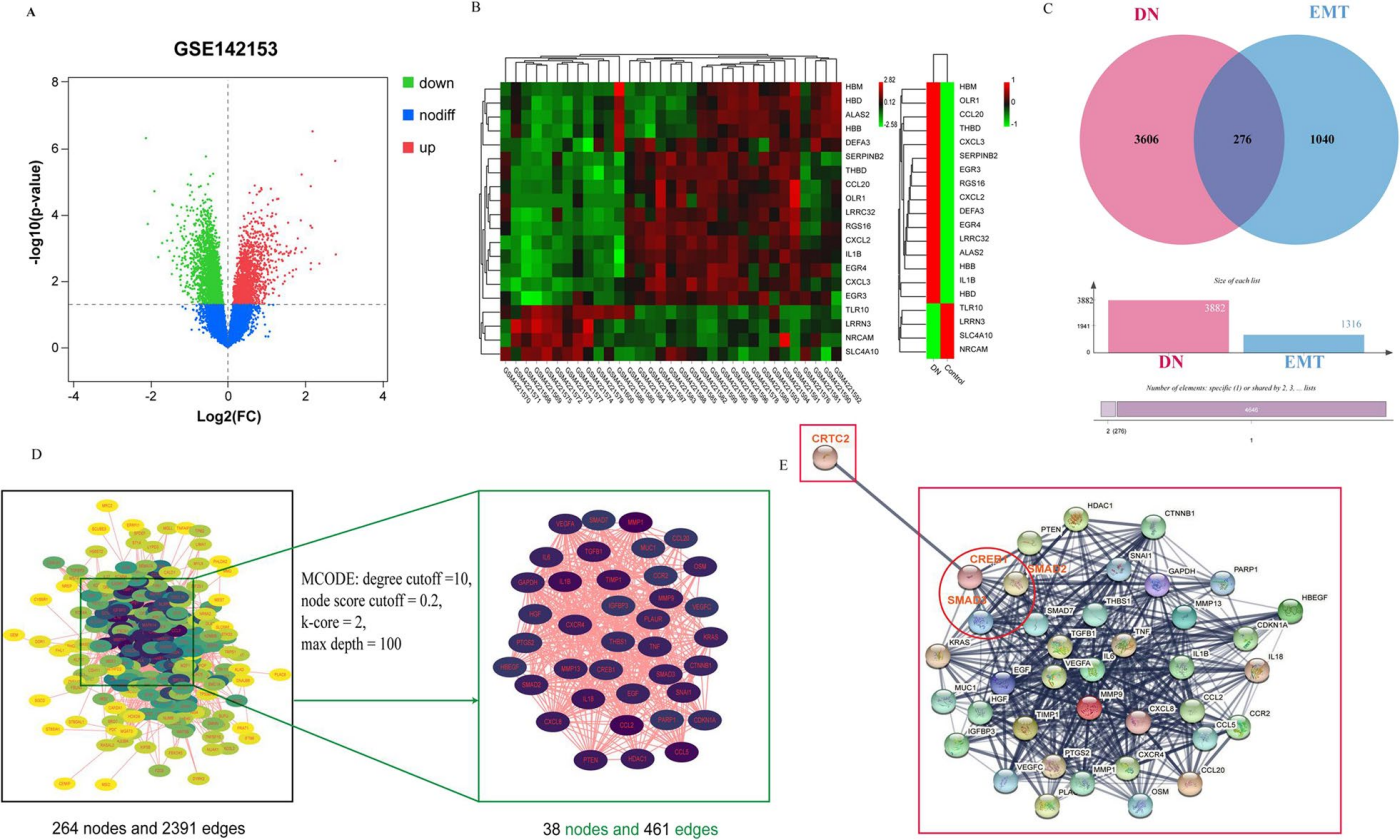
Bioinformatics analysis: **A** volcano map: P-value to draw a volcano map after correction. The red dots in the figure indicate genes that are significantly up-regulated, and green dots indicate genes that are significantly down-regulated; **B** heat map: a heat map of differential gene expression, where different colors represent the expression trends in different samples; **C** intersection of differentially expressed genes in DN patients and EMT-related genes; **D** topological analysis of DN-EMT related genes; **E** the protein interaction network of the potential relationship between CRTC2 and DN-EMT-related core targets

A total of 1316 ERGs were identified with the EMT gene database and molecular feature database v7.1. The limma package in the R package was used to analyze the GSE142153 dataset to identify DEGs in DN (P < 0.05), including 1772 highly expressed genes and 2450 genes with low expression (Fig. 1A, B). The DEGs were mapped with the ERGs to identify the DEGs during EMT in DN, and the results included 157 highly expressed genes and 202 genes with low expression (Fig. 1C).

### PPI analysis

ERGs-DN was imported into the STRING database to generate a PPI network composed of 317 nodes and 2587 edges. Subsequently, the core PPI network module was obtained using MCODE, and it consisted of 38 nodes and 461 edges (Fig. 1D). Then, the core module gene and CRTC2 gene were imported into the STRING database, and it was found that the combined score of CRTC2 and CREB1 in the core module gene was 0.999. This result indicated that CRTC2 may be potentially related to ERGs-DN. The Smad pathway is currently recognized as the most important signal transduction pathway in the process of renal tubular epithelial cell transdifferentiation. Smad signal transduction occurs as follows (Yao et al. 2019). After being stimulated by upstream stimulation factors, Smad2 and Smad3 are activated by phosphorylation and bind to Smad4 to form heteropolymers, which are then translocated to the nucleus and act on specific target genes to exert biological effects. Figure 1E shows that CREB1 is potentially related to Smad2 and Smad3. Therefore, we speculate that CRTC2 plays an important role in promoting the occurrence of diabetic renal interstitial fibrosis and that CRTC2 promotes the transdifferentiation of diabetic renal tubules through the CREB-Smad2/3 signaling pathway.

### Establishment of a mouse model of DKD

**Fig. 2:**
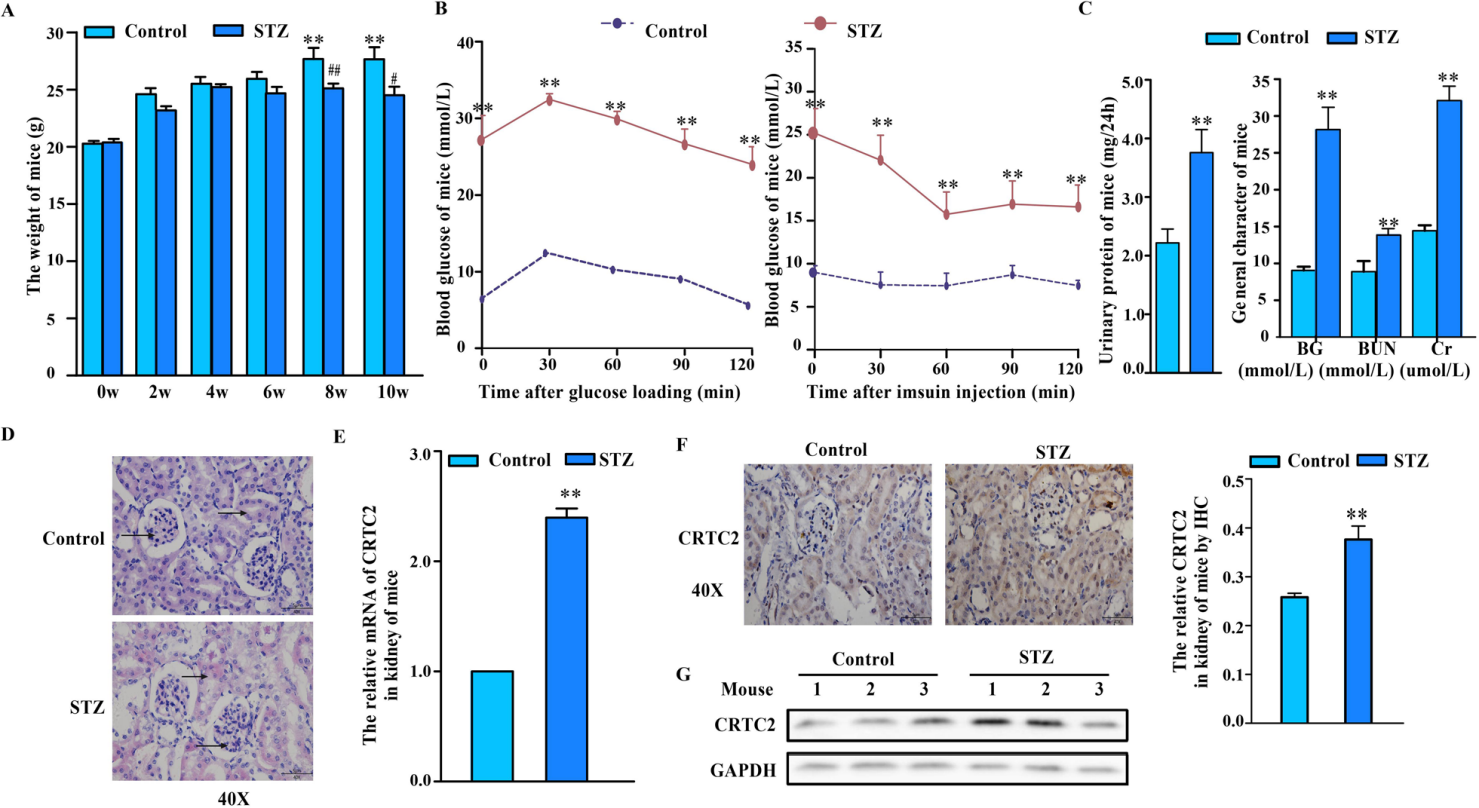
Abnormal CRTC2 were expressed in kidney of DKD mice. C57BL/6J mice were intraperitoneally injected with STZ (30 mg/Kg) or PBS control. The STZ mice were fed with high fat diet and control mice were fed with normal diet. **A** The mice were weighed (g) every 2 weeks. **B** OGTT and ITT were performed on the mice one week before death (n = 6–9). **C** 24 h urinary protein were measured after 10 weeks of treatment. Serum Fasting blood glucose (FBG), blood urea nitrogen (BUN) and serum creatinine (SCr) level (n = 6–9). **D** H&E staining in kidney tissue sections from mice. **E** CRTC2 were detected by real-time PCR in the kidney of mice (n = 6–9). **F** Immunohistochemistry analysis of CRTC2 in kidney tissue sections from mice and quantitative analysis were conducted by Image J software. **G** CRTC2 was detected by Western blot in the kidney of mice. The data were presented as means ± SD. The statistical significance was relative to the STZ group *p < 0.05, **p < 0.01

Control mice showed good growth status, had normal dietary habits, were alert, exhibited active reactions, and had shiny hair. However, STZ-treated mice exhibited gradually worsening symptoms of diabetes, including polyphagia, polyuria, and polydipsia, and they had fluffy and white hair and exhibited lazy and bored behavior. In addition, the weight of the STZ-treated mice decreased significantly compared with that of the control mice (Fig. 2A). We observed biochemical indicators of the mice at different time points. In the OGTT and ITT, the STZ-treated mice exhibited significantly abnormal glucose levels (Fig. 2B left) and insulin resistance (Fig. 2B right) compared with the control group. The 24-h urine protein levels of the STZ group were significantly higher than those of the control group (Fig. 2C). We also found that the fasting blood glucose (FBG), serum urea nitrogen (BUN) and creatinine (SCr) levels were increased in STZ-treated mice (Fig. 2C). Compared with the control mice, the STZ-treated mice exhibited glomerular swelling and hypertrophy, obvious proliferation of glomerular mesangial cells, hyperplasia and sclerosis of the mesangial matrix, and obvious vacuoles in the cytoplasm of renal tubular epithelial cells (Fig. 2D). These phenomena showed that our model was successfully established.

### Abnormal CRTC2 expression was observed in the kidneys of DKD model mice

To explore the expression of CRTC2 in mice with diabetic kidney disease, we measured the expression of CRTC2 in mice with diabetic kidney disease and control mice. The results showed higher mRNA levels of CRTC2 in the kidneys of STZ-treated mice compared with control mice (Fig. 2E), and similar results were obtained by immunohistochemical staining (Fig. 2F) and Western blotting analysis (Fig. 2G). The results showed that CRTC2 expression was elevated in the kidneys of mice with diabetic kidney disease.

### CRTC2 modulated EMT in vivo and in vitro

**Fig. 3:**
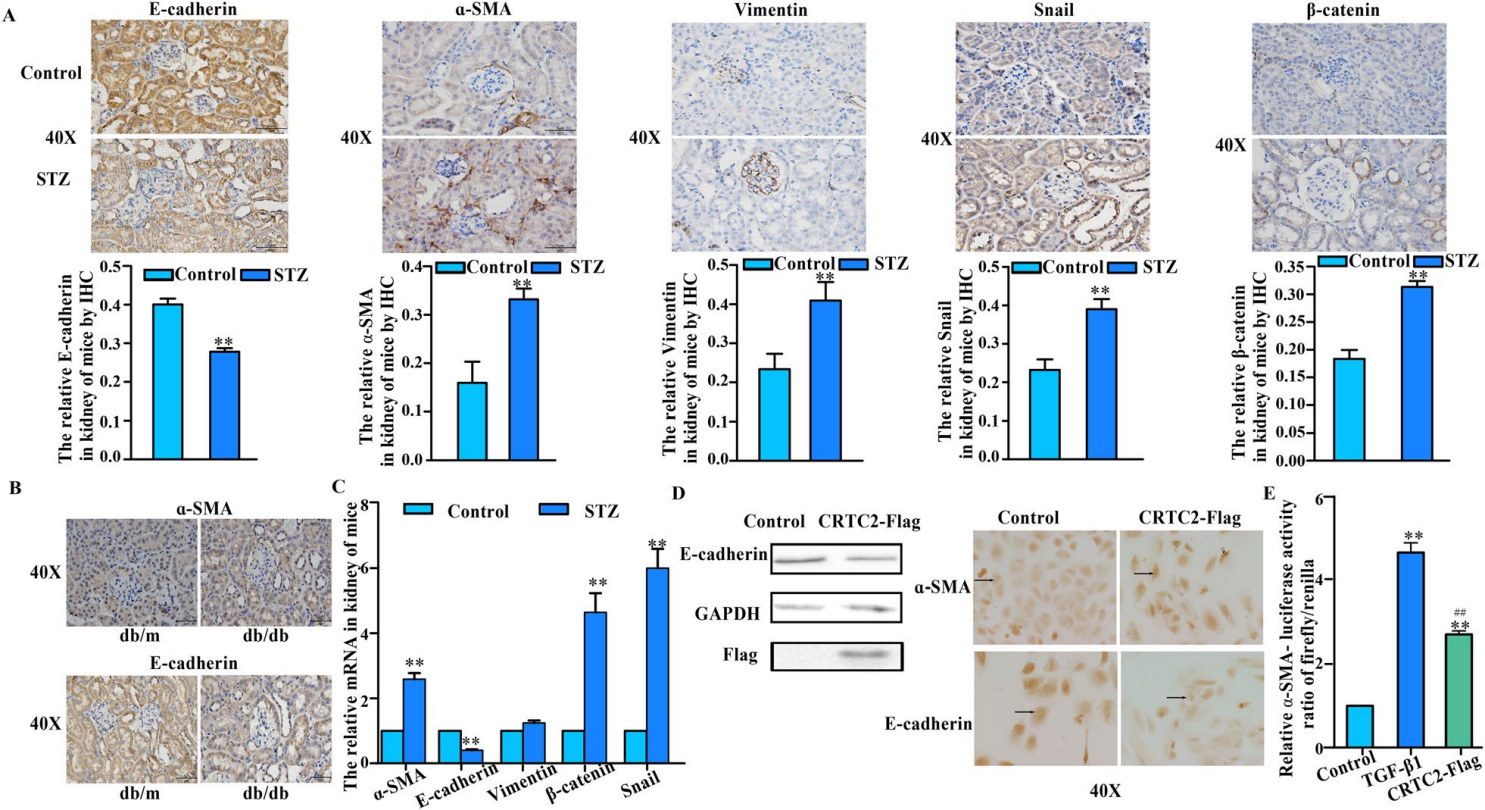
CRTC2 modulated EMT in vivo and in vitro. C57BL/6J mice were intraperitoneally injected with STZ (30 mg/Kg) or PBS (control). The STZ mice were fed with high fat diet and control mice were fed with normal diet. **A** E-cadherin, α-SMA, Vimentun, Snial and β-catenin were detected by immunohistochemistry in the kidney of mice. **B** E-cadherin and α-SMA were detected by immunohistochemistry in the kidney of db/db mice. **C** Genes of kidney were detected by real-time PCR (n = 3). **D** E-cadherin was detected by Western blot (left), and E-cadherin and α-SMA were detected by immunohistochemistry (right) in HK-2 cells with overexpressed CRTC2-Flag plasmid for 48 h. **E** HK-2 cells were transfected with α-SMA luciferase constructs with or without control vector, as well as CRTC2 overexpression plasmid (CRTC2-Flag) for 48 h. The cells were treated with TGF-β1 (8 ng/ml, 48 h). Luciferase activity was assayed in triplicate. **p < 0.01 compared with Control group. ^##^p < 0.01 compared with TGF-β1 group

Immunohistochemical staining showed that E-cadherin expression (E-cadherin downregulation is known as a key event in EMT (Sommariva and Gagliano 2020) expression was suppressed in STZ mice kidneys (Fig. 3A). Moreover, the expression levels of the epithelial-mesenchymal transition (EMT) marker α-SMA (Zhang et al. 2019) were increased (Fig. 3A) in STZ-treated mouse kidneys. We also observed that other EMT markers, such as Vimentin, Catenin, and Snail, were increased in the kidneys of STZ-treated mice according to immunohistochemical staining (Fig. 3A). We also observed that the expression of E-cadherin and α-SMA was similar in db/db mice (Fig. 3B), which are used as a spontaneous model of type 2 diabetes (Pelletier et al. 2020). The real-time PCR results were similar to the immunohistochemical staining results (Fig. 3C).

Furthermore, we found that overexpression of CRTC2 significantly reduced the expression of E-cadherin in HK-2 cells (Fig. 3D left) and that the expression of α-SMA was increased in HK-2 cells overexpressing CRTC2 (Fig. 3D right). To further elucidate the role of CRTC2 in EMT, we conducted a luciferase assay. Specifically, we designed a luciferase reporter α-SMA transcription for use in this assay. We found that CRTC2 overexpression caused significantly higher α-SMA luciferase activity than the control group, and similar results were obtained in the TGF-β1 group (Fig. 3E). These data showed that CRTC2 was involved in the EMT process.

### CRTC2 promoted EMT through CREB-Smad2/3 signaling pathway

**Fig. 4:**
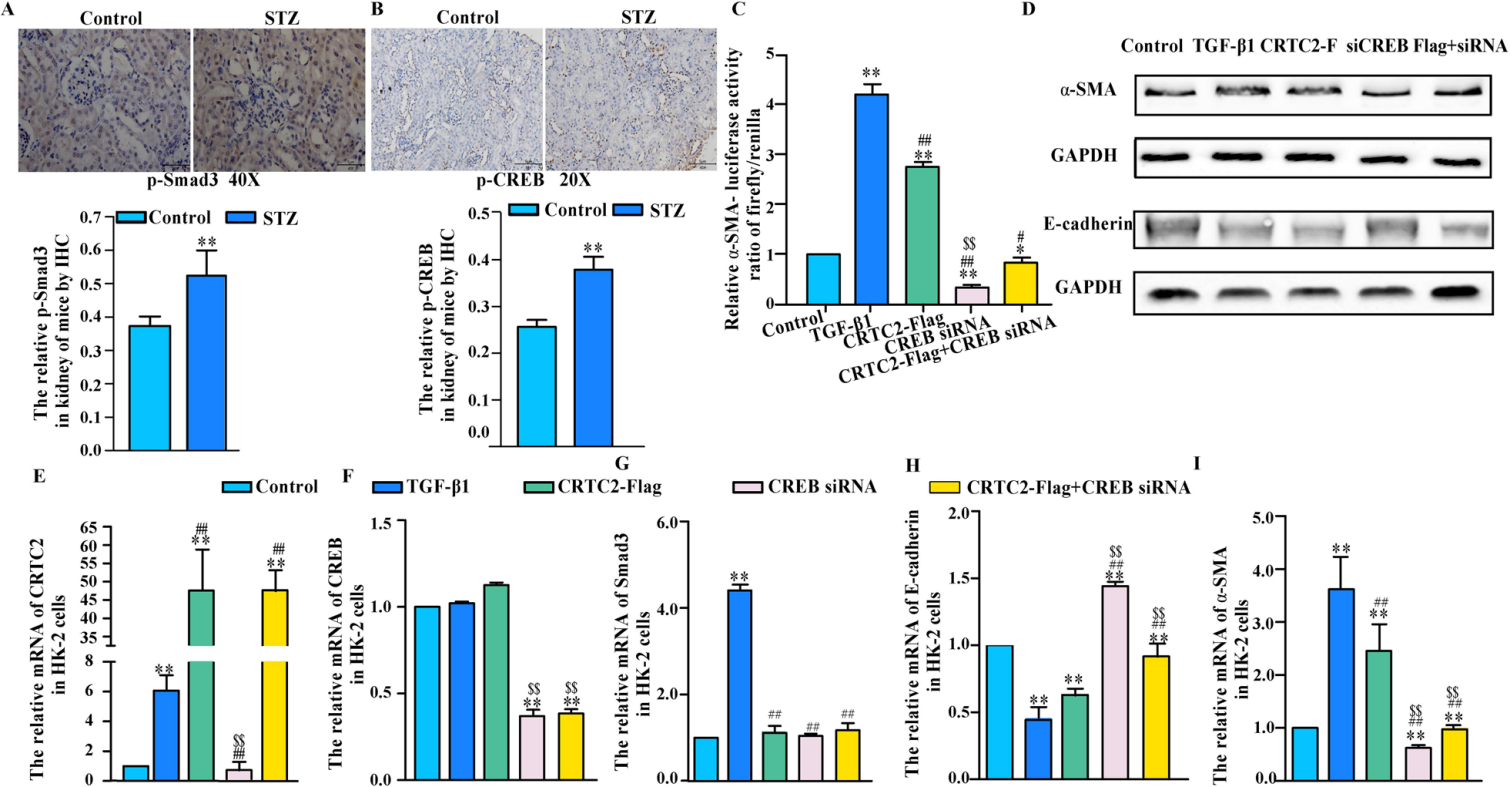
CRTC2 promoted EMT through CREB-Smad2/3 signaling pathway. C57BL/6 J mice were intraperitoneally injected with STZ (30 mg/Kg) or PBS (control). The STZ mice were fed with high fat diet and control mice were fed with normal diet. p-Smad3 (**A**) and p-CREB (ser133) (**B**) were detected by immunohistochemistry in the kidney of mice. **C** HK-2 cells were transfected with α-SMA luciferase constructs with or without control vector, as well as CRTC2 overexpression plasmid (CRTC2-Flag) or SiRNA CREB for 48 h. The cells were treated with TGF-β1 (8 ng/ml, 48 h). Luciferase activity was assayed in triplicate. **p < 0.01 compared with Control group. ^#^p < 0.05 compared with TGF-β1 group, ^##^p < 0.01 compared with TGF-β1 group. ^$$^p < 0.01 compared with CRTC2-Flag group. **D**–**I** HK-2 cells were transfected with or without control vector, as well as CRTC2 overexpression plasmid (CRTC2-Flag) or SiRNA CREB for 48 h. And cells were treated with TGF-β1 (8 ng/ml, 48 h). Protein expression were detected by Western blot (**D**). Gene expression was analyzed by real-time PCR (**E**–**I**). All experiments was assayed in triplicate (**E**–**I**), *p < 0.05,**p < 0.01 compared with control group. ^#^p < 0.05, ^##^p < 0.01 compared with TGF-β1 group. ^$$^p < 0.01 compared with CRTC2 overexpression plasmid group

Next, we explored the relationship between the EMT process and CRTC2, CREB, and Smad2/3. First, immunohistochemical staining showed higher levels of p-Smad3 and p-CREB expression in STZ-treated mouse kidneys than in control mouse kidneys (Fig. 4A, B). Furthermore, we conducted a luciferase assay in HK-2 cells. In the luciferase experiment, we found that the α-SMA luciferase activity was significantly higher than that in the control group, and similar results were obtained in the TGF-β1 group after CRTC2 overexpression. We also found that the CRTC2-induced increase in α-SMA luciferase activity was attenuated after silencing CREB or Smad2/3 (Fig. 4C, 5A). These results showed that the EMT process in diabetic kidney disease is closely related to CRTC2, CREB and Smad2/3.

**Fig. 5:**
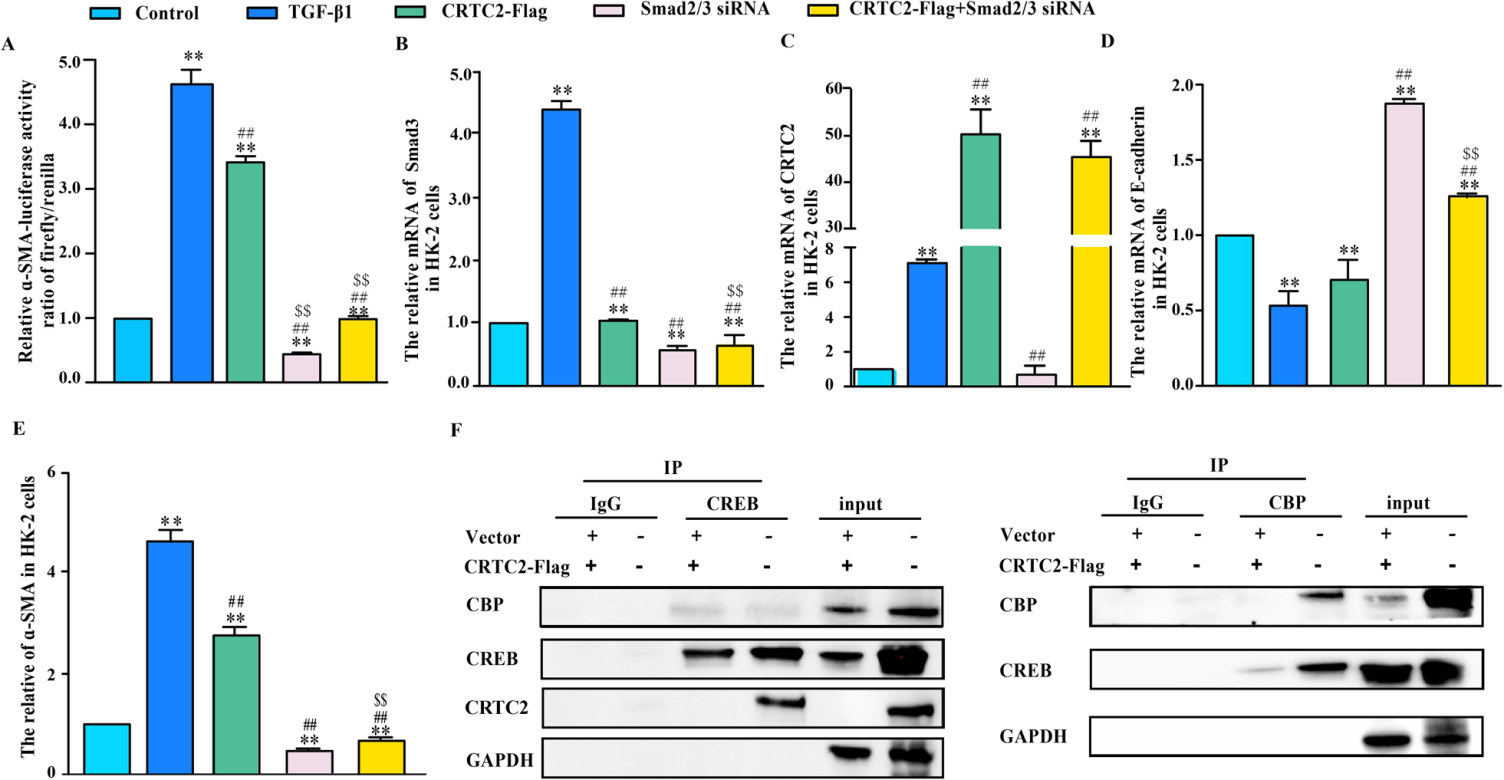
CRTC2 promoted EMT through CREB-Smad2/3 signaling pathway. HK-2 cells were transfected with or without control vector, as well as CRTC2 overexpression plasmid or SiRNA Smad2/3 for 48 h. And cells were treated with TGF-β1 (8 ng/ml, 48 h). **A** α-SMA promoter activity was detected using dual luciferase reporters in triplicate. **B**–**E** Smad3, CRTC2, E-cadherin and α-SMA gene expression was analyzed by real-time PCR. **F** 293T cells were transfected with overexpression plasmid of CRTC2 (CRTC2-Flag) for 48 h, immunoblots of co-immunoprecipitation assay showing binding of CBP and CREB in cells. All experiments was assayed in triplicate, **p < 0.01 compared with Control group. ^##^p < 0.01 compared with TGF-β1 group. ^$$^p < 0.01 compared with CRTC2 overexpression plasmid group

cAMP-regulated transcriptional coactivators (CRTCs) are a family of CREB coactivators. In this study, we found that the increase in α-SMA expression caused by the overexpression of CRTC2 was alleviated after transfection of siRNA targeting CREB, and the opposite results were obtained for E-cadherin expression in HK-2 cells according to Western blotting (Fig. 4D). The mRNA level of CRTC2 was increased by TGF-β1 stimulation; however, there was no change after silencing CREB (Fig. 4E). We also found that the mRNA level of CREB was not changed after the overexpression of CRTC2 in HK-2 cells (Fig. 4F). Then, we measured the mRNA expression of Smad3 in HK-2 cells. The results showed that the Smad3 mRNA levels were slightly changed in the CRTC2-overexpressing group and the CREB-silenced group (Fig. 4G). Furthermore, we measured the mRNA levels of E-cadherin and α-SMA in HK-2 cells. We found that the mRNA expression of E-cadherin was decreased in the CRTC2-overexpressing group, but the change was attenuated after silencing CREB (Fig. 4H). The mRNA level of α-SMA showed the opposite trend (Fig. 4I).

Additionally, we investigated whether the change in E-cadherin and α-SMA expression in HK-2 cells was caused by CRTC2 via Smad2/3. We found that the CRTC2-induced increase in α-SMA luciferase activity was attenuated after silencing Smad2/3 (Fig. 5A). The results showed that the mRNA level of Smad3 was increased by TGF-β1 stimulation, but there was no change after CRTC2 overexpression (Fig. 5B). It is likely that CRTC2 has no effect on the mRNA expression of Smad3, and silencing Smad2/3 did not affect the mRNA expression of CRTC2 (Fig. 5C). Additionally, we observed that the mRNA expression of E-cadherin was decreased after TGF-β1 stimulation and CRTC2 overexpression, and this decrease was mitigated by silencing Smad2/3 (Fig. 5D). The mRNA level of α-SMA exhibited the opposite trend (Fig. 5E). In this study, we also found that CBP:CREB complex formation was increased in 293T cells overexpressing CRTC2 compared with the control group in CO-IP experiments (Fig. 5F).

## Discussion

**Fig. 6:**
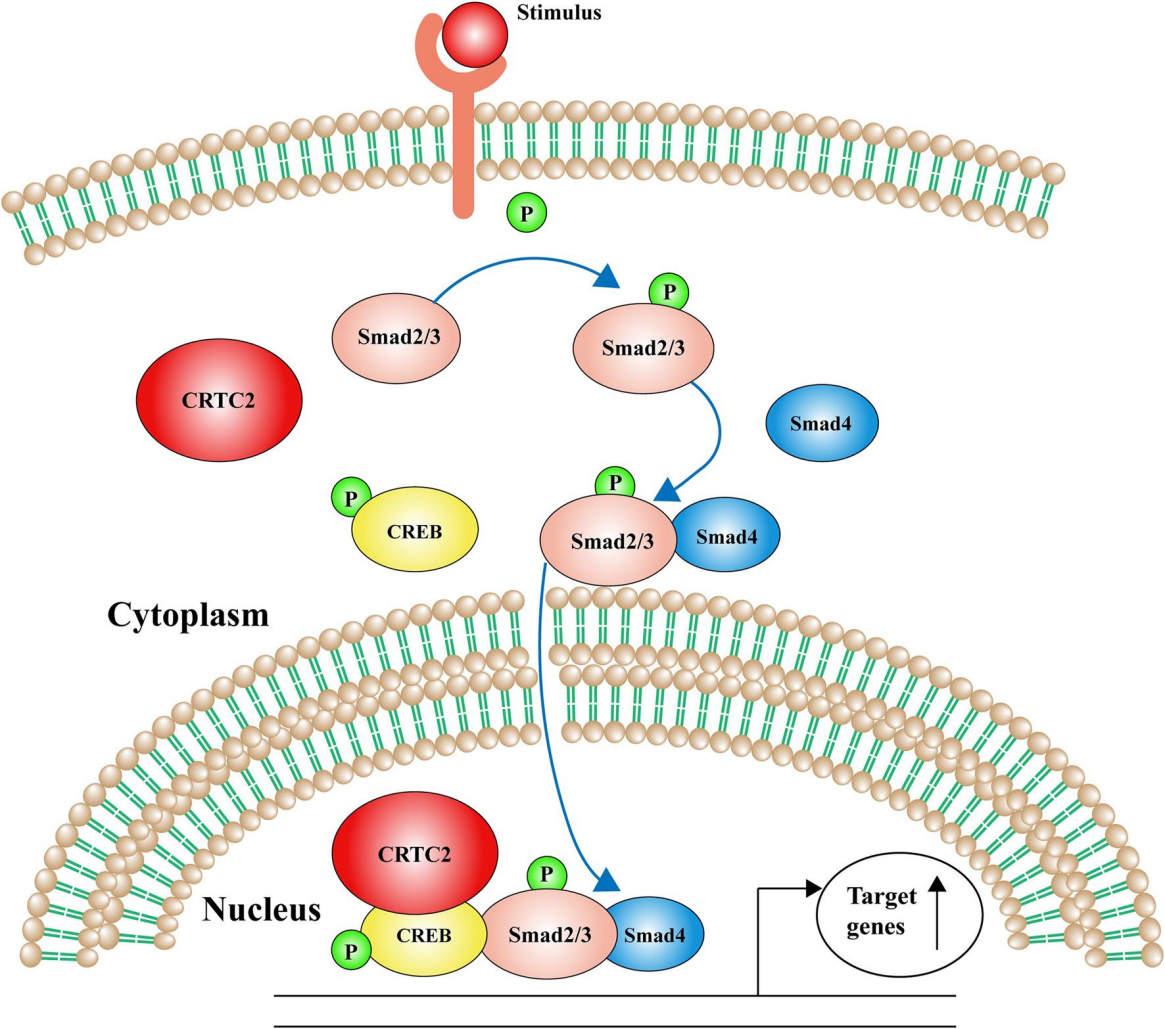
CRTC2 modulates EMT via CREB-Smad2/3 pathway in DKD. CRTC2, as a co-activator of CREB, enters into nucleus and binds to CREB, then CREB promotes the Smads complex to identify the target genes to induce EMT process

DKD is one of the main microvascular complications of diabetes, and it is an important cause of death among diabetic patients; Additionally, disordered glucose and lipid metabolism can exacerbate diabetic kidney disease (Tziomalos and Athyros 2015). To investigate whether glycolipid coregulatory factors are involved in the pathogenesis of DKD, we first explored the effect of CRTC2, a coregulator of both lipid metabolism and glucose metabolism, on the EMT process in DKD and its underlying molecular mechanism via bioinformatics analysis and experimental verification. We found that CRTC2 expression is elevated in DKD, and CRTC2 may promote the transdifferentiation of diabetic renal tubular epithelial cells through the CREB-Smad2/3 signaling pathway, leading to renal interstitial fibrosis (the related mechanism is shown in Fig. 6).

In this study, we observed a significant increase in CRTC2 expression in DKD. Studies have shown that CRTC2 is continuously activated by glycosylation as blood sugar continuously increases, which leads to a continuous increase in blood sugar levels and disorders of glucose metabolism (Koo et al. 2005). CRTC2 acts as a coactivator of CREB, which is a member of the basic leucine zipper transcription factor family, and can mediate various cAMP-dependent transcription pathways in metabolic tissues (Lee et al. 2018). After phosphorylation, CREB interacts with CREB-binding protein and affects the expression of genes that are downstream of cAMP-responsive element (CRE), such as genes encoding the rate-limiting enzyme in gluconeogenesis (Wang et al. 2020) and factors related to cholesterol synthesis (Li et al. 2017a). Gluconeogenesis contributes to DKD. Previous studies have shown that insulin resistance is an early change that is observed in CKD, and is it worsened by ongoing chronic inflammation (Alicic et al. 2022), CRTC2 significantly contributes to the development of insulin resistance, and disruption of CRTC2 increases insulin sensitivity (Wang et al. 2010b; Hogan et al. 2015).

We first confirmed the connection between CRTC2 and the EMT process in DKD as well as the underlying molecular mechanism. EMT is an important process in the pathogenesis of renal tubulointerstitial fibrosis and involves the loss of epithelial cell characteristics (loss of E-cadherin expression) and an increase in the expression of mesenchymal cell markers (such as α-SMA). TGF-β1 is considered to be the most effective mediator of EMT and renal fibrosis (Wang et al. 2015). After TGF-β1 binds to its receptor, serine/threonine kinase is activated and induces Smad2/Smad3 phosphorylation, and then phosphorylated Smad2/3 binds to Smad4 and migrates to the nucleus, where this complex regulates the transcription of target genes responsible for EMT; this mechanism has been widely accepted (Jia et al. 2015).

CREB is activated by phosphorylation and participates in the pathogenesis of multiple metabolic pathways and organ fibrosis. The phosphorylation of the Ser133 site of CREB can activate embryonic fibroblasts to secrete a-SMA (Li et al. 2016). During kidney EMT, phosphorylated Smad2/3 binds to common Smad4 to form a Smad complex, which translocates into the nucleus to regulate the transcription of target genes, including Smad7 (Yao et al. 2015). We found a potential relationship between CREB and Smad2/3 through the STRING database. After CREB phosphorylation increases (e.g., in response to elevated intracellular cAMP levels), CREB-binding protein (CBP) effectively binds to p-CREB, thereby enhancing its transcriptional activity (Cardinaux et al. 2000). p-CREB is further acetylated, and CREB activity is prolonged in the late stage of attenuation (Wang et al. 2010a). On the other hand, CBP is known to directly interact with Smad protein complexes and act as a Smad transcription coactivator (Chen et al. 2007), and as a broad-spectrum coactivator, CBP directly interacts with R-Smad-Smad4 through the C-terminal domain to activate the transcription of TGF-β1-response genes (Chen et al. 2007). Therefore, the competition between cAMP-dependent CREB/CBP and TGF-β-dependent Smad/CBP binding for binding with the coactivator CBP is considered to be the mechanism by which Smad-dependent signal transduction is inhibited (Wójcik-Pszczoła et al. 2020). In this study, we confirmed the potential relationship between CREB and Smad2/3 via bioinformatics and experimental verification. However, the more specific mechanism underlying this relationship needs to be further explored.

CRTC2 is a coactivator of CREB that promotes the activity of CREB (Wang et al. 2010b).CREB stimulates targeted gene expression at promoters that contain CREs. These typically appear as either palindromic (TGACGTCA) or half-site (TGACG or CGTCA) sequences (Altarejos and Montminy 2011). In this study, we also observed that CRTC2 increased ɑ-SMA-luciferase activity. However, we did not explore the regions of CRTC2 or CREB that are necessary for regulating the ɑ-SMA promoter. Further experimental studies are required to elucidate these potential regions that regulate the ɑ-SMA promoter.

## Conclusions

In summary, we first predicted the potential molecular mechanism by which the glucose and lipid metabolism factor CRTC2 participates in the EMT process in diabetic kidney disease with bioinformatics tools. Subsequently, our experiments showed that CRTC2 can stimulate the EMT process in vivo and in vitro. Moreover, we proved that CRTC2 partially activates the EMT process through the CREB-Smad2/3 signaling pathway in diabetic renal tubules.

### Supplementary Information

**Additional file 1. **EMT-related genes

## Data Availability

All data generated or analyzed during this study are included in this article. The raw data supporting the conclusions of this article will be made available by the authors, without undue reservation.

## Abbreviations

ɑ-SMA: ɑ-Smooth muscle actin
CREB: CAMP response element-binding protein
CRTC2: Cyclic adenosine monophosphate-responsive element (CRE) binding protein-regulated transcription coactivator (CRTC)2
DKD: Diabetic kidney disease
EMT: Epithelial-to-mesenchymal transition
ESRD: End-stage renal disease
FBG: Fasting blood glucose
OGTT: Oral glucose tolerance test
STZ: Streptozotocin
